# Proteomic Analysis of the Air-Way Fluid in Lung Cancer. Detection of Periostin in Bronchoalveolar Lavage (BAL)

**DOI:** 10.3389/fonc.2020.01072

**Published:** 2020-07-03

**Authors:** Yangying Zhou, Weiming Yang, Minghui Ao, Naseruddin Höti, Edward Gabrielson, Daniel W. Chan, Hui Zhang, Qing Kay Li

**Affiliations:** ^1^Department of Pathology, Johns Hopkins Medical Institutions, Baltimore, MD, United States; ^2^Department of Oncology, Sidney Kimmel Cancer Center at Johns Hopkins Medical Institutions, Baltimore, MD, United States

**Keywords:** lung cancer, proteomic analysis, *N*-glycoprotein, bronchoalveolar lavage, periostin expression, ELISA

## Abstract

**Background:** Bronchoalveolar lavage (BAL) is a specific type of air-way fluid. It is a commonly used clinical specimen for the diagnosis of benign diseases and cancers of the lung. Although previous studies have identified several disease-associated proteins in the BAL, the potential utility of BAL in lung cancer is still not well-studied. Based upon the fact that the majority of secreted proteins are glycoproteins, we have profiled *N*-glycoproteins in BAL collected from lung cancers, and investigated the expression of glycoproteins such as the matrix *N-*glycoprotein, periostin, in lung cancers.

**Methods:** BAL specimens (*n* = 16) were collected from lung cancer patients, and analyzed using mass spectrometry-based quantitative *N*-glycoproteomic technique. Additional BAL specimens (*n* = 39) were independently collected to further evaluate the expression of periostin by using an enzyme-linked immunosorbent assay (ELISA).

**Results:** A total of 462 glycoproteins were identified in BAL samples using *N*-glycoproteomic technique, including 290 in lung adenocarcinoma (ADC, *n* = 5), 376 in squamous cell carcinoma (SQCC, *n* = 4), 309 in small cell lung carcinoma (SCLC, *n* = 4), and 316 in benign lung disease (*n* = 3). The expressions of several glycoproteins were elevated, including 8 in ADC, 12 in SQCC, and 17 in SCLC, compared to benign BALs. The expression of periostin was detected in all subtypes of lung cancers. To further investigate the expression of periostin, an ELISA assay was performed using additional independently collected BALs (*n* = 39) The normalized levels of periostin in benign disease, ADC, SQCC, and SCLC were 255 ± 104 (mean ± SE) and 4,002 ± 2,181, 3,496 ± 1,765, and 1,772 ± 1,119 ng/mg of total BAL proteins.

**Conclusion:** Our findings demonstrate that proteomic analysis of BAL can be used for the study of cancer-associated extracellular proteins in air-way fluid from lung cancer patients.

## Introduction

Lung cancer is a heterogeneous group of tumors, including non-small cell carcinoma (NSCLC) and small cell carcinoma (SCLC). In the NSCLC, squamous cell carcinoma (SQCC) and adenocarcinoma (ADC) are the two dominant subtypes (1, 2). The recent large scale of genetic studies, including the International Cancer Genome Consortium (ICGC) and The Cancer Genome Atlas (TCGA), have identified multiple driver gene mutations in NSCLC (3, 4). These studies have greatly improved our molecular understanding of lung cancers and facilitated the development of targeted drugs and immunotherapies (3–9). However, despite these advancements, lung cancer remains one of the leading causes of cancer-associated mortality in the United States and worldwide (1, 2). Clinically, the majority of lung cancer patients present with the advanced-stage disease; thus, it is critical to improve detection and monitor disease progression in order to improve the overall survival of lung cancer.

Currently, we commonly use the combination of imaging and histological assessment of the lesion for the diagnosis of lung cancer. Several types of specimens can be used for histomorphological evaluation, such as bronchial brushing and bronchoalveolar lavage (BAL) samples, and transbronchial fine-needle aspiration (EBUS-TBNA) biopsy with or without ultrasound guidance specimens (10–13). In addition, biomolecular markers in the blood, bronchial epithelium, and sputum are also being tested (14, 15). Studies have shown that the use of glycoproteins could potentially improve the accuracy and specificity of lung cancer diagnosis (16, 17).

The air-way fluid bronchoalveolar lavage (BAL) contains proteins released from lung parenchyma that may be useful for the detection and monitory of certain benign lung diseases, as well as lung cancer progression (14–16, 18). In our previous study of lung ADC, we found that certain glycoproteins are differentially expressed between cancer and benign BAL using the solid-phase extraction of *N*-glycoprotein (SPEG) and liquid chromatography-tandem mass spectrometry (LC-MS/MS) (16). The study showed that glycoproteins could be detected and quantified in BAL fluids, supporting that the glycoproteomic analysis of BAL could be useful for the identification of extracellular cancer-associated proteins, especially when the specimens did not qualify the criteria to have enough cells for histological examination (16–18). Therefore, analysis of *N*-glycoproteins in the air-way fluid of lung cancers has a potential utility for the identification of cancer-associated glycoproteins.

Studies have shown that tumor microenvironment, i.e., desmoplastic stroma, plays a critical role in tumor progression, invasion, and metastasis (19, 20). Several desmoplastic stroma *biomarker*s have been characterized in cancers (19, 20). Among these stromal biomarkers, periostin stands out in playing a remarkable role during cancer progression (21). Periostin is an extracellular matrix *N-*glycoprotein in the desmoplastic stroma around solid tumors, and is involved in the regulation of extracellular matrix (ECM) network (21). It interacts with other ECM proteins, such as collagen V, fibronectin, and tenascin-C to form an extracellular environment, in which cells can interact with each other to promote growth and survival. Periostin also interacts with various cell-surface receptors, particularly with integrins, through the PI3K/AKT signaling pathway to promote epithelial-mesenchymal transition (EMT), cell growth, and tumor angiogenesis and invasion (22, 23).

The expression of periostin in the desmoplastic stroma of the tumor has been associated with the aggressiveness of tumor behavior, increased metastatic potential, and advanced tumor stages in multiple cancer types, such as head and neck (24), colon (22), breast (25), lung (23, 26–30), and others (31). Previously, we have identified that the expression of periostin in the stroma of prostate cancer is associated with aggressive clinical behavior of the tumor (32). Furthermore, periostin is an extracellular protein and accessible via the bloodstream, as such, it has been considered to be a promising therapeutic target for cancer patients (31). In lung carcinoma, the expression of periostin has been linked to tumor invasiveness and metastasis (23, 26–30). Recent studies have also shown that periostin can be detected in the serum of NSCLC patients (29, 30), indicating the possible utility of periostin as a cancer-associated glycoprotein in lung cancer. However, the functional significances of *N*-glycoproteins expression in lung cancers, particularly periostin expression in air-way fluid, is still poorly understood.

In this study, we investigated the profile of *N*-glycoproteins in BAL specimens and identified periostin to be significantly overexpressed in different subtypes of lung cancers. These findings were further validated using an independent cohort of BAL specimens by enzyme-linked immunosorbent assay (ELISA).

## Materials and Methods

### Collection of Lung BAL Specimens

The discarded BAL specimens were collected from the cytologic laboratory after the evaluation of lung cells. Briefly, 5–10 cc normal saline was applied to rinse the lung during the bronchoscopic procedure, the bronchoscopy was used to collect the fluids into a sterilized test tube and sent to the cytological laboratory. After carefully examined the pulmonary cells, the BAL fluids of ADC (*n* = 5), SQCC (*n* = 4), SCLC (*n* = 4), and benign (*n* = 3) were used for the proteomic analysis. For a validation study, additional BAL specimens were collected for the ELISA assay, including six cases of benign lung diseases, 18 cases of primary lung ADCs, 6 cases of primary lung SCLCs, and 9 cases of primary lung SQCCs. To remove the cellular debris, all BAL specimens were centrifuged and were then stored at −80°C freezer before the analysis.

This study was approved by the Institution Review Board (IRB) of Johns Hopkins Medical Center.

### Peptide Extraction From BAL Specimens

All the BAL specimens were sonicated on an ice bath for 5 min and then centrifuged at 12,600 rpm for 10 min. One-hundred microliters of trifluoroethanol (TFE) was added into each specimen and incubated for 2 h. The protein concentration of the BAL samples was calculated using the BCA protein assay kit (Thermo Fisher Scientific Inc., Rockford, IL). Then the proteins were reduced by Tris (2-carboxyethyl) phosphine (TCEP) (Pierce, Rockford, IL) at room temperature for 1 h. BAL specimens were incubated with the sequencing grade trypsin (Promega, Madison, WI) at 37°C overnight. The digested peptides were used for further analysis.

### Capture of *N-*Glycopeptides

As previously described, the SPEG method was used to capture *N*-linked glycopeptides (16). C18 column (Waters, Sep-Pak Vac) was used for peptide desalting, and tryptic peptides were eluted using 80% of acetonitrile (ACN)/0.1% trifluoroacetic acid (TFA), oxidized with sodium periodate at a final concentration of 10 mM, and incubated for 1 h in the dark at room temperature. The oxidation reaction was quenched using sodium sulfite at a final concentration of 60 mM for 10 min at room temperature. The samples were coupled to hydrazide resin (Bio-Rad, Hercules, CA) in 80% ACN and 0.1% TFA for 4 h at room temperature. Then the resin was washing three times by using 100 mM NH_4_HCO_3_, 1.5 M NaCl, and H_2_O to remove non-glycosylated peptides. *N*-linked glycopeptides were released from the resin by mixing with 1 μl PNGase F (New England Biolabs, Ipswich, MA) in 100 mM NH_4_HCO_3_ at 37°C overnight. After the C18 cartridge, the purified glycopeptides were dried by Speed-Vac and resuspended with 0.4% acetic acid for further use of LC-MS/MS analysis.

### Liquid Chromatography-Tandem Mass Spectrometry (LC-MS/MS) Analysis

Peptides were analyzed by LC-MS/MS on an Orbitrap Velos (Thermo Fisher Scientific, USA) and a nanoLC system (Eksigent, Dublin, USA). One microgram of peptides was subjected to a self-packed C18 column with a 120 min gradient. Mobile phase flow rate was set as 300 nL/min with 0.2% FA (A) and 0.2% FA, 95% ACN (B), respectively. The de-glycosylated peptides were separated with gradient elution of phase B from 5%, and ramped to 30% in 86 min. LC-MS/MS data acquisition used a data-dependent analysis (DDA) method of the top 10 most abundant ions. The MS spectra were collected from 350 to 1,800 *m/z* at a resolution of 70,000 followed by data-dependent higher-energy collisional dissociation (HCD) MS/MS, with a 2 *m/z* isolated mass window.

### Protein Identification and Label-Free Quantification

Proteome Discoverer software (Thermo Fisher Scientific, USA) was applied for protein annotation. The dataset was searched against a human protein database (the International Protein Index human protein database containing a total number of 91,464 entries, version 3.87). The MS and MS/MS tolerances were set as 20 ppm and 0.06 Da, respectively. For de-glycosylated peptides identification, the cysteine residues were modified by carbamidomethyl group (C, +57.105 Da) as a stable modification, and methionine oxidation (M, +15.9949 Da) and asparagine deamidation (N, +0.98 Da) were set as dynamic modifications. A maximum of 2 miss cleavages was allowed. Then we used SIEVE software (Thermo Scientific, USA) for the label-free, semi-quantitative analysis of glycopeptides and glycoproteins. The chromatograms of raw files were aligned after importing into the SIEVE. Frame parameters were set as follows: mass range from 350 to 1,800, retention time from 0 to 120 min, a maximum frame of 100,000, frame *m/z* width of 10 ppm, frame retention time width for 2.5 min, the intensity threshold of 5 ×10^5^. After the SIEVE framing, the results from Proteome Discoverer were filtered with 1% false-discovery rate (FDR) and quantified using the unique peptide for a protein. In SIEVE analysis, the frames for formal glycopeptides (i.e., those containing NXS/T, X≠P, and N was deamidated) were targeted for quantification. Data were normalized by total ion current (TIC).

### Enzyme-Linked Immunosorbent Assay (ELISA)

In the assay, 100 μl of BAL specimens were incubated with periostin capture antibody in a 96-well-microtiter plate overnight at 4°C, and then incubated with HRP-conjugated mouse anti-human periostin antibody at 4°C for 30 min. Tetramethylbenzidine was added and interacted at room temperature for 20 min. Then using an absorbance reader (Biotek, Winooski, VT) to acquire the absorbance readings at 450 nm. Recombinant human periostin (concentration range: 0–250 ng/mL) was used to generate a standard curve from a four-parameter logistic curve fit. All samples were analyzed duplicated and calculated the average of the two values. The total protein concentration of all BAL samples was determined using the BCA protein assay. The levels of periostin were expressed as ng/mg total proteins in BAL specimens.

### Statistical Analysis

Characteristics of clinicopathological information were evaluated using the SPSS statistical software (version 24.0, SPSS Inc., USA) The χ^2^ and Fisher's exact tests were used to calculate the *P*-value. The relationship between periostin and pathological characteristics of NSCLS patients was using Spearman rank correlation analysis. All tests were determined using bilateral 95% confidence intervals (CI). If the alpha value was <0.05, it was considered statistically significant (*P* <0.05).

## Results

### Clinical Information

Discarded BAL fluids were collected from the cytologic laboratory for the proteomic analysis, including ADC (*n* = 5), SQCC (*n* = 4), SCLC (*n* = 4), and benign lung diseases (*n* = 3). All cancer cases were primary lung cancers. For 16 proteomic BAL samples, the median age of patients was 63 years, ranging from 23 to 88 years; and the female and male ratio was: 1:1. In 13 cancer cases, 1 case was pT1, 3 cases were pT2, 5 cases were pT3, and 4 cases were pT4. The smoking status revealed that six patients were non-smokers, and 10 were former or current smokers (Supplementary Table 1).

Additional BAL specimens were collected for the ELISA assay, including six cases of benign lung diseases, 18 cases of primary lung ADCs, 6 cases of primary lung SCLCs, and 9 cases of primary lung SQCCs. Of 39 cases, the median age of patients was 65 years, ranging from 28 to 85 years. The female and male ratio was: 1.3:1. In 33 cancer cases, 4 cases were pT1, 10 cases were pT2, 12 cases were pT3 and 7 cases were pT4. The smoking status revealed that eight patients were non-smokers, and 31 were former or current smokers (Supplementary Table 2).

### Detection of Glycoproteins and Periostin in BAL Specimens by Label-Free Quantification and SIEVE Analysis

**Figure 1:**
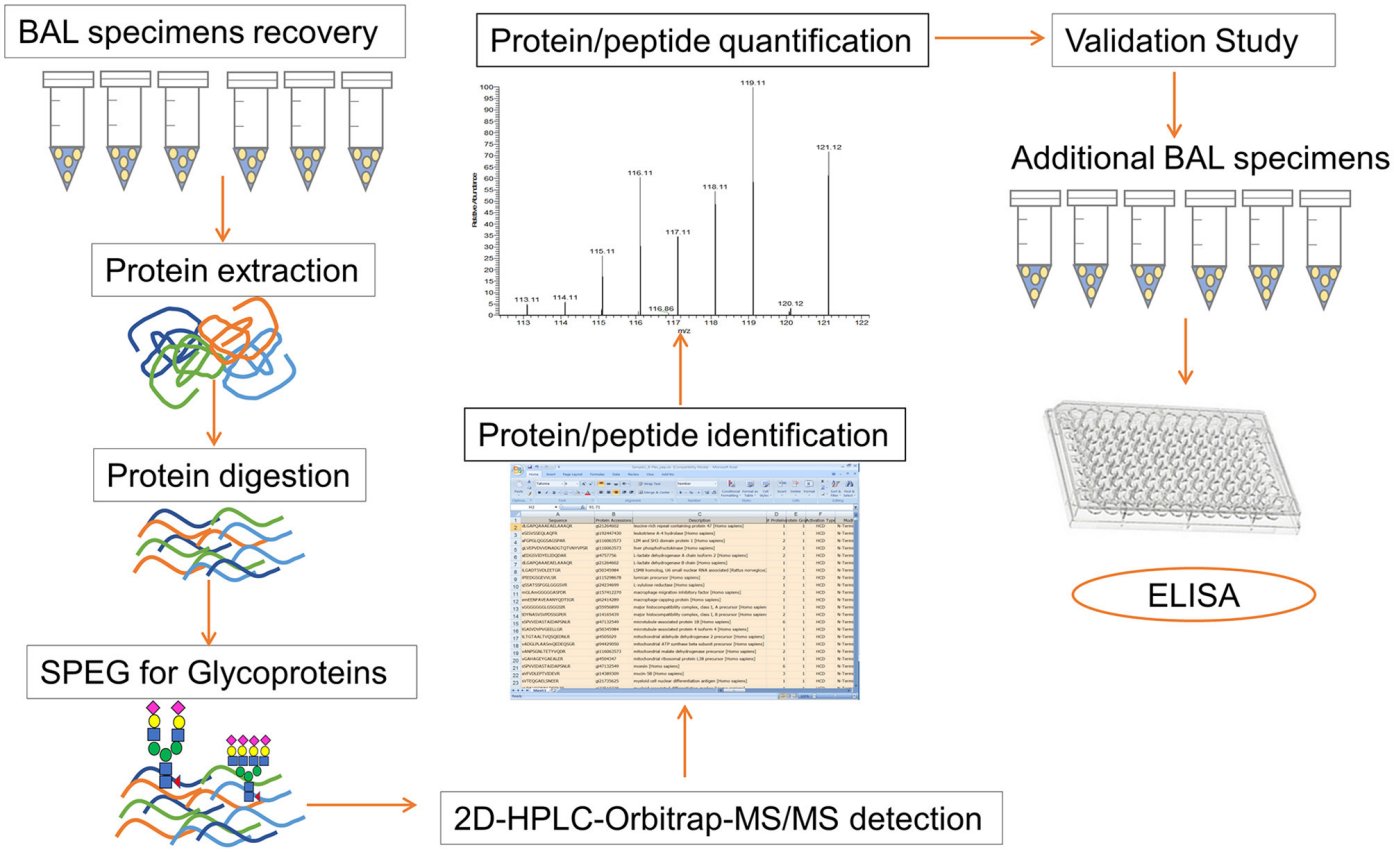
Workflow for the proteomics detection in BAL specimen and subsequent validation study. The overall strategy of our study consists of following steps: (1) recovery of peptides from BAL samples, (2) capture of formerly *N*-linked glycopeptides using SPEG method, (3) Orbitrap Velos LC-MS/MS analysis, (4) identification and quantification of protein/peptide using Proteome Discoverer and SIEVE software, (5) analysis and comparison of the *N*-glycoprotein abundance in different BAL specimens, and (6) validation studies by using an ELISA assay.

The overall strategy in our study included the following main steps: (1) recovery of peptides from BAL samples, (2) capture of formerly *N*-linked glycopeptides using the SPEG method, (3) analysis of the *N*-glycoprotein abundance in different BAL specimens, and (4) validation of our findings using an ELISA assay (Figure 1).

**Figure 2:**
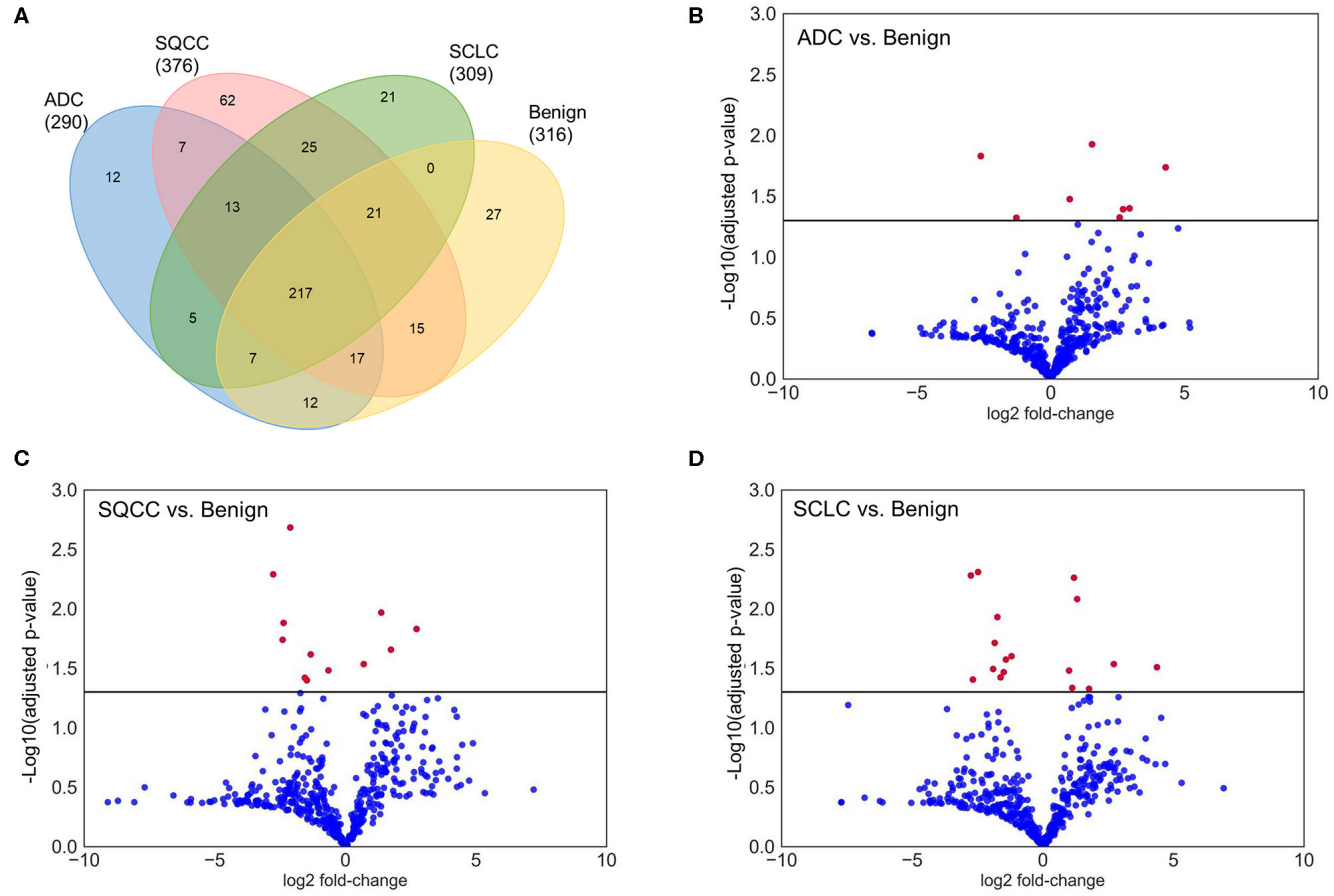
Overview identification and quantification of glycoproteins in BAL samples. **(A)** Venn diagram of identified glycoproteins in lung ADCs, SQCCs, SCCs, and benign samples. **(B)** Volcano plot showing the variance in protein expression between lung ADCs and benign samples. **(C)** Volcano plot showing the variance in protein expression between lung SQCCs and benign samples. **(D)** Volcano plot showing the variance in protein expression between lung SCLCs and benign samples (Wilcoxon signed-rank test, Red dot: adjusted *P* <0.05, Blue dot: adjusted *P* ≥ 0.05).

In total, 462 glycoproteins from 1,081 glycopeptides were identified in BAL samples, whereas, there were 290, 376, 309, and 316 glycoproteins identified in ADCs, SQCCs, SCLCs, and benign samples, respectively. Compared to the benign samples, there were 8, 12, and 17 glycoproteins differentially expressed in ADCs, SQCCs, and SCLCs, respectively (adjusted *P* <0.05, Wilcoxon signed-rank test). The differentially expressed glycoproteins included periostin (POSTN), vascular cell adhesion protein 1 (VCAM1), TNF Receptor Superfamily Member 19 (TNFRSF19), and mucin-13 (MUC13). Overall data are summarized in Figure 2 and Supplementary Table 3.

**Figure 3:**
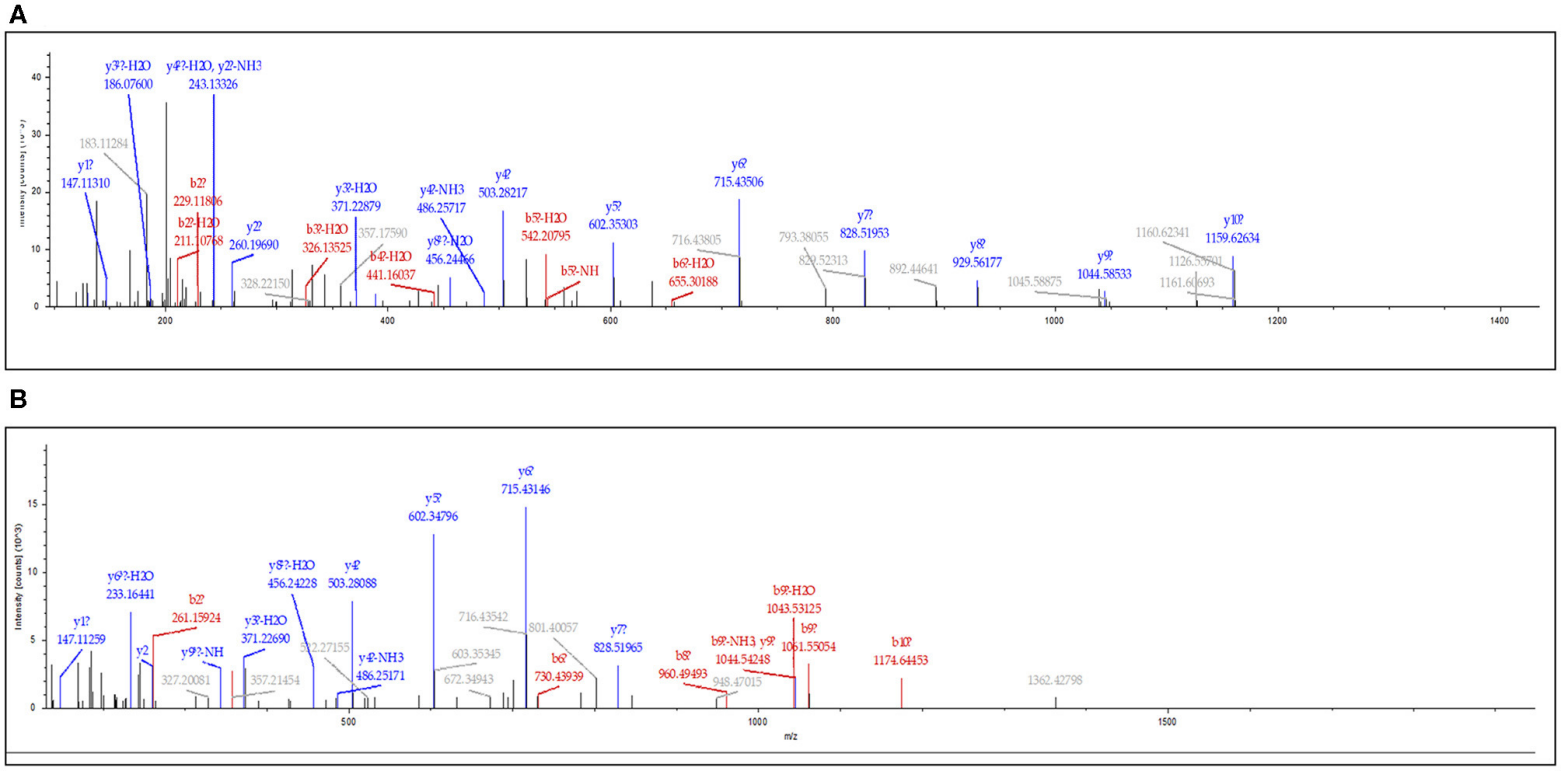
Identification of periostin in lung BAL samples. The MS/MS spectrum corresponding to periostin peptide sequence EVN*DTLLVNELK (Charge: +2, Monoisotopic m/z: 694.37050 Da, MH+: 1,387.73373 Da) **(A)** and IFLKEVN*DTLLVNELK (Charge: +3, Monoisotopic m/z: 630.35892 Da, MH+: 1,889.06221 Da) **(B)**. N* is the formerly *N*-linked glycosylation site and deaminated to Aspartic Acid (D) when *N*-glycans were released from the site by PNGase F.

**Figure 4:**
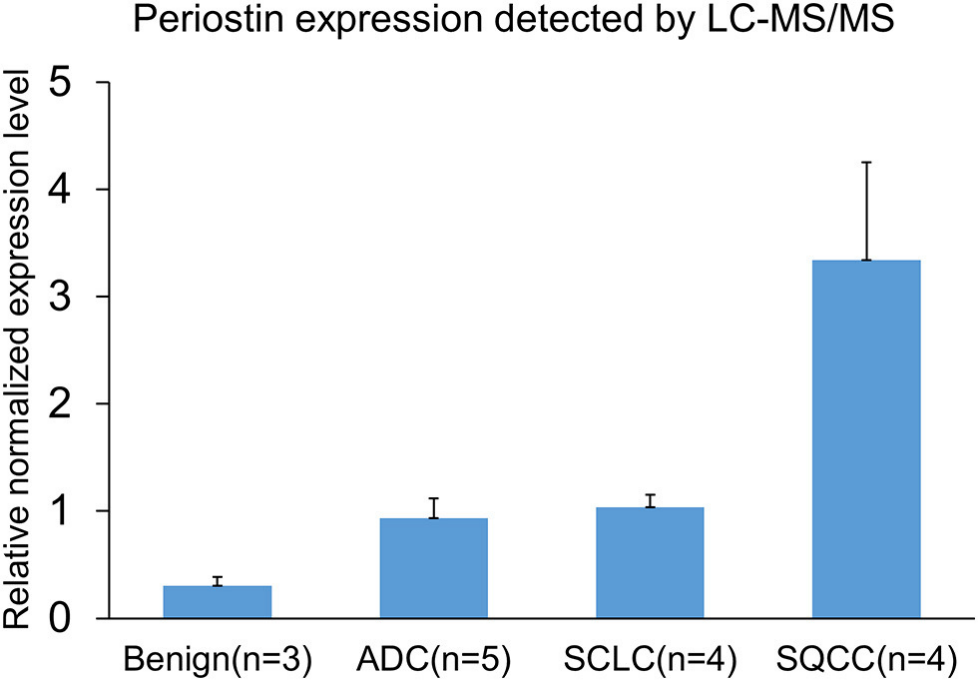
Detection and quantification of periostin in BALs by mass-spectrometry (MS) and SIEVE software. Data are shown as relatively normalized expression levels (means ± SE). The mean values of periostin in benign disease, ADCs, SQCCs, and SCLCs were 0.30 ± 0.08, 0.94 ± 0.18, 3.34 ± 0.91, and 1.04 ± 0.12.

Among these differentially expressed glycoproteins in lung cancer, periostin was a particularly interesting one. The periostin was identified in the BAL samples using a combination of *N*-glycopeptides SPEG capture with SIEVE label-free quantification. Two glycopeptides of periostin were detected with the sequences of EVN^*^DTLLVNELK and IFLKEVN^*^DTLLVNELK (Figure 3). The mean values (the relative normalized expression level) of periostin in benign disease, ADCs, SQCCs, and SCLCs were 0.30 ± 0.08 (mean ± SE), 0.94 ± 0.18, 3.34 ± 0.91, and 1.04 ± 0.12 (Figure 4). By comparing to the benign samples, we detected higher expressions of periostin in all three tumor types.

### Validation of Periostin Expression in BAL by an ELISA Assay

**Figure 5:**
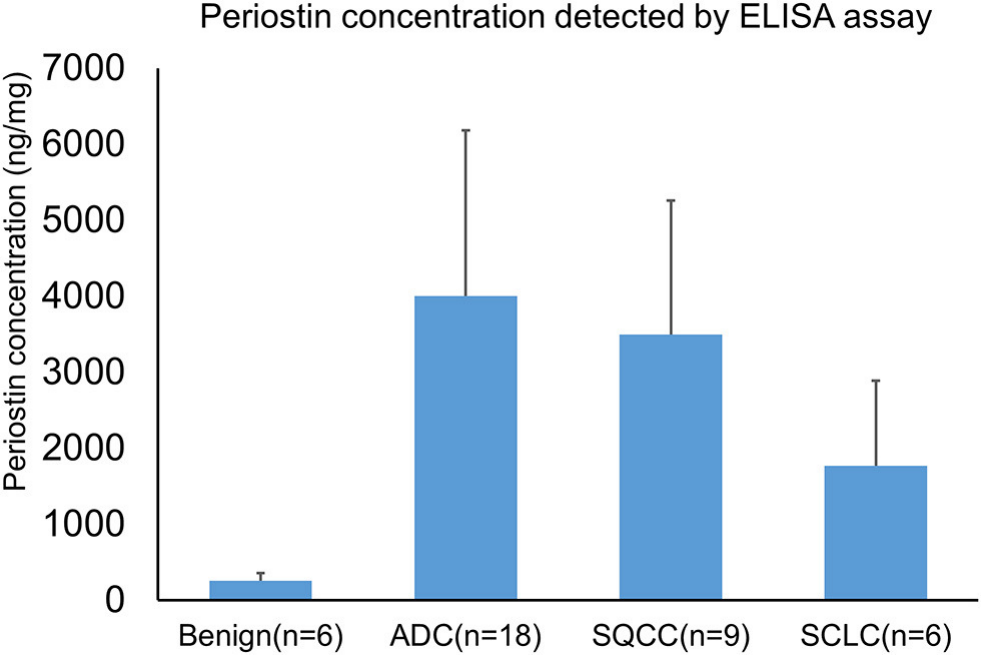
Detection of periostin in BAL specimens by an ELISA assay. BAL specimens were independently collected from benign lung disease and lung cancer patients. A total of 39 BAL specimens were used for the assay. The protein concentration of each specimen was determined and used for the normalization of periostin levels. The mean expression levels of periostin in benign disease, ADCs, SQCCs, and SCLCs were 255 ± 104 (mean ± SE) and 4,002 ± 2,181, 3,496 ± 1,765, and 1,772 ± 1,119 ng/mg of total BAL proteins.

We further investigated the expression of periostin in BAL using an independently collected cohort of lung cancer patients by an ELISA assay. In the assay, we included six cases of benign lung diseases, 18 cases of primary lung ADCs, 9 cases of primary lung SQCCs, and 6 cases of lung SCLCs. The protein concentration of individual specimens was also determined and used for the normalization of periostin levels. The average expression levels of periostin in benign disease, ADCs, SQCCs, and SCLCs were 255 ± 104 (mean ± SE) and 4,002 ± 2,181, 3,496 ± 1,765, and 1,772 ± 1,119 ng/mg of total BAL proteins (Figure 5). The periostin levels elevated in all cancer groups compared to the benign lung lesion group. These findings were in consistent with the LC-MS/MS data. However, we also found differential expression trends among lung cancer types.

## Discussion

Glycoproteins play a critical role in the regulation of cellular biological functions as well as in the discovery of cancer-associated proteins (33, 34). Glycosylation, especially the *N-*linked glycosylation, is commonly identified on a large number of cellular proteins, including cell surface proteins, transmembrane proteins, and extracellular proteins secreted in body fluids (33, 35). Studies have shown that glycoproteins secreted in body fluids such as urine, serum, cerebrospinal fluid (CSF), and BAL can be analyzed to monitor the disease progression in benign lung disease. These characteristics make glycoproteins as an indispensable group of potential cancer-associated proteins in the realm of clinical and biological research of cancers. We studied the profile of glycoproteins in the air-way fluid using the solid-phase extraction of *N*-glycoprotein and mass spectrometry-based LC-MS/MS approach, and then, validated our findings by using additional independent collected BAL specimens and ELISA assay. This is the first study utilizing air-way fluid to characterize *N*-glycoproteins expression in different types of lung cancer patients, particularly as proof-of-principle how BAL samples can be used for the study of cancer-associated extracellular glycoproteins in lung cancers.

In our study, a total of 462 glycoproteins were identified in the BAL samples, including 290, 376, 309, and 316 glycoproteins identified in ADCs, SQCCs, SCLCs, and benign samples, respectively. Compared to the benign samples, there were 8, 12, and 17 glycoproteins differentially expressed in ADCs, SQCCs, and SCLCs, respectively, such as POSTN, VCAM1, TNFRSF19, and MUC13.

VCAM-1 (CD106) is a 90-kDa glycoprotein, and frequently expressed in endothelial cells, which is participated in the regulation of cellular adhesion and inflammation-related activities (36, 37). The expression of VCAM-1 is associated with the activation of inflammatory cytokines, such as TNFα and ROS, and is relevant to the progression of several immunological disorders and cancers (38, 39). MUC13 is a cell surface mucin glycoprotein and plays a critical role in cancer cell proliferation and apoptosis (40, 41). It is often over-expressed in various cancer types, such as colorectal (42), gastric (43), pancreatic (44), and ovarian cancers (45). Some studies have also indicated its role in tumorigenesis by activating Wnt and NF-κB signaling pathways (46, 47). TNFRSF19, also known as TROY, is the member of TNF receptor superfamily, and usually transduces cytokine signals through specific adaptor proteins that bind to the intracellular domain (ICD). High expression of TNFRSF19 is related to the inferior prognosis in melanoma (48), glioblastoma (49), and colorectal cancers (50), and is reported promoting tumorigenesis via TGFβ signaling pathways in nasopharyngeal carcinoma (51). We identified the differential expression of these proteins in the lung cancer BALs compared to the benign controls, which provided new insights for the discovery of lung cancer-associated glycoproteins. However, the role of these proteins still needs to be further investigated in lung cancers.

In addition to VCAM-1, MUC13, and TNFRSF18, periostin is a particularly interesting one. Periostin is a 93 kDa matrix *N-*glycoprotein, that was first identified in osteoblast-like cells. It is present in many healthy tissues and involved in bone formation, cardiac development, wound healing, and other cellular functions (19–21). Periostin is also known to play a significant role in tumorigenesis via the promotion of EMT of carcinoma cells and the regulation of the integrin signaling pathways, which leads to subsequent activation of the AKT/PKB (protein kinase B) and the FAK (focal adhesion kinase) network (19–24). A recent study has also suggested that the expression of periostin in fibroblasts may be involved in the process of premetastatic niche formation in the lung tissue (52). In lung cancer, the expression of periostin has been linked to tumor invasive and metastasis (23, 26–30). Recent studies have also shown that periostin can be detected in the serum of NSCLC patients (29, 30), indicating the potential utility of periostin as a therapeutic target in lung cancer. In an animal study using periostin knock-out mice, it has been demonstrated that the expression of periostin is involved in the phosphorylation of ERK in the Akt and FAK pathways (28). Furthermore, periostin is an extracellular protein and should be accessible via air-way fluid as well as other extracellular fluid. Recent studies have also demonstrated that the expression of periostin can be detected in the pleural effusions of NSCLC patients (53).

Quantitative proteomic analysis of BAL fluid has been applied to study certain benign lung diseases such as cystic fibrosis, asthma, interstitial lung disease (14, 15) and idiopathic eosinophilic pneumonia (54), but the protein profile in different types of lung cancers, especially the *N*-linked glycoproteomics, has not been well-studied. We detected the glycoprotein expression in BAL from all subtypes of lung cancers, including both small cell and non-small cell lung cancers. More importantly, we found that periostin could be detected in air-way fluids by glycoproteomic analysis, which was validated using an independently collected cohort of BAL samples. Our finding is also in accordance with the observation of elevated serum levels of periostin in lung cancer (29). Taken together, our study suggests that secreted periostin from tumor cells can be detected in the air-way fluid, indicating that it may play a tumorigenic role in lung cancer development.

In this study, we first analyzed *N*-glycoproteins including periostin expressions in lung cancer BALs by using MS-based analysis, and then, further validated our findings by ELISA assay. The sequential steps of our analysis provide strong evidence that the air-way fluid can be used for the development of a clinical assay for the detection of cancer-associated glycoproteins in personalized medicine. In the MS-based analysis, we found that the periostin was differentially expressed among all lung cancer types, with a relatively higher level in SQCC using the *N*-glycoprotein capture technique. Although we found a similar result in the subsequent ELISA assay among lung cancer types, the expression of periostin revealed a relatively higher level in ADC. Our finding of variable expression of periostin in lung cancers may be due to several factors, such as different detection techniques used in the assay and the relatively small sample size in our study. The role of periostin in lung cancers needs to be further investigated.

Our study has several strengths, we have revealed that mass spectrometry-based *N*-linked glycoprotein analysis of air-way fluid could be used for the identification of cancer-associated glycoproteins in lung cancer, most notably that it is the first study to characterize the glycoproteins as well as periostin expression in different types of lung cancer patients using BAL samples. It may provide valuable information for lung cancer patients. However, our study also has limitations, such as the relatively small sample size and the large standard deviation during analysis, particularly in the ELISA assay. These limitations indicate that the sample collections procedure needs to be standardized prior to the proteomic analysis and the validation study. Further efforts to improve the detection of cancer-associated glycoproteins in the air-way fluid and large cohorts of validations are required for our future study.

In summary, our study utilizes an unique approach for the detection of glycoproteins in lung air-way fluids and demonstrates that various proteins, including periostin, are differentially expressed in BAL in lung cancer patients. Our findings provide new insight into the biological role of periostin in lung cancers. Although our findings indicate the possibility that periostin may be a potential prognostic marker in lung cancer patients, the further large-scale study is still needed to validate our findings. As current research continues to focus on the study of the mechanism and prognostic significance of periostin, these data may contribute to refine our knowledge of periostin as a cancer-associated glycoprotein and lead to a better understanding its role in cancer biology.

## Data Availability Statement

The datasets presented in this study can be found in online repositories. The names of the repository/repositories and accession number(s) can be found at: MassIVE database, https://massive.ucsd.edu/ProteoSAFe/static/massive.jsp. Accession: MSV000085469.

## Ethics Statement

This study was reviewed by the Institutional Review Board of the Johns Hopkins Medical Institution. Ethical approval was not required for the study on human participants in accordance with the local legislation and institutional requirements. Written informed consent was not required to participate in this study in accordance with the national legislation and the institutional requirements.

## Author Contributions

YZ, WY, HZ, and QL: conceptualization. YZ, MA, EG, and QL: project administration and methodology. YZ, WY, MA, and NH: data curation and software. YZ, WY, and HZ: formal analysis. MA and NH: validation. YZ and NH: writing—original draft. EG, DC, HZ, and QL: writing—review and editing. All authors read and approved the final manuscript.

## Conflict of Interest

The authors declare that the research was conducted in the absence of any commercial or financial relationships that could be construed as a potential conflict of interest.
